# MgO-Lignin Dual Phase Filler as an Effective Modifier of Polyethylene Film Properties

**DOI:** 10.3390/ma13030809

**Published:** 2020-02-10

**Authors:** Karol Bula, Grzegorz Kubicki, Teofil Jesionowski, Łukasz Klapiszewski

**Affiliations:** 1 Institute of Materials Technology, Faculty of Mechanical Engineering, Poznan University of Technology, PL-60965 Poznan, Poland; 2 Institute of Chemical Technology and Engineering, Faculty of Chemical Technology, Poznan University of Technology, PL-60965 Poznan, Poland

**Keywords:** lignin, MgO-lignin hybrid materials, polyethylene, polymer film, welding

## Abstract

Functional magnesium oxide-lignin hybrid materials were obtained via mechanical grinding. Their particle shape and size as well as physicochemical properties were characterized. MgO-lignin materials with biocomponent content (between 20% and 80% amount of total weight of filler) were used as a partially bio-structured modifier of low density polyethylene. The composites with 5% by weight of dual fillers and polyethylene grafted with maleic anhydride were compounded in a twin screw extruder working in co-rotating mode. The prepared blends were cast extruded using a single screw extruder and laboratory cast line. The properties of the obtained films were verified in case of their weldability. The seal strength as well as shear test and tear strength of the welded sheets were examined. The results showed that the shortest equivalent time required to perform correct weld occurred in the system, where the highest amount of lignin was used in hybrid filler MgO-L (1:5 *w*/*w*). From mechanical tests of welds, a sharp increase in ultimate seal force was noticed for almost all compositions with lignin, especially where MgO was coupled with a high lignin content. For those composition seal open force raised up to 37.0 N, from the value of 23.6 N, achieved for neat low density polyethylene (LDPE). Tear strength of weld sheets confirmed once more that LDPE composition with MgO-L (1:5 *w*/*w*) achieved the highest ultimate force with its value of 71.5 N, and it was ~20.0 N higher than in the case of neat LDPE.

## 1. Introduction

Lignin is a biodegradable, carbon neutral component which absorbs the ultraviolet (UV) radiation of sunlight, and is available at low cost and in huge volumes as a by-product of the paper industry [1,2]. A literature survey revealed that current studies regarding the use of lignin are focused on the production of lignin-based thermoplastic composites. Studies on the addition of the lignin into a non-biodegradable material are take the initiation of partial biodegradability of the obtained composites into consideration. The idea results from the known relation, that the properties of thermoplastics may be markedly influenced by the incorporation of bio-organic fillers, especially by a low cost product such as lignin [3,4,5,6,7,8]. This improvement is offered when the lignin content is at an optimum ratio and/or pro-adhesive additives are used in parallel. Due to the hydrophilic nature of lignin, the pro-adhesive agent is obligatory, for example in polyolefin/lignin systems. In such triple component materials, a significant shift of selected mechanical properties is observed [9,10,11,12]. 

Due to the low miscibility of pure lignin with polyolefins, problems such as lignin agglomeration and phase separation occur very often. Polyethylene-grafted-maleic anhydride (MAPE), which improves the compatibility between the low density polyethylene matrix and lignin, reduced the interfacial tension between components in immiscible blends. Such effects of MAPE could be attributed to the new bonds formed between maleic anhydride group-grafted LDPE and the OH groups of lignin. On the other hand, there is a good compatibility between the LDPE and the polyethylene (PE) matrix segments of MAPE [13,14,15].

Our previous study also confirmed the very positive role of kraft lignin on selected properties of high density polyethylene (HDPE) and LDPE, in case of which lignin were combined with oxides (SiO_2_, ZnO) and incorporated into the polymer matrices in the presence of maleated polyethylene [16,17].

On the other hand, utilization of lignin into polyolefin could be associated with the ability of lignin to absorb UV. In general, the chemical structure of lignin has different crosslinked phenolic structures, bonded together by ether linkages and carbonic structures. The guaiacyl and syringyl phenolic units of lignin are responsible for its antioxidant activity [18,19]. Since the phenolic OH groups of lignin are able to scavenge free radicals, numerous trials were conducted to use it as a stabilizer in order to improve the thermal oxidative stability of PE. Most of these attempts were successful and confirmed the antioxidant effect of lignin in the system with linear low density polyethylene (LLDPE), LDPE, and HDPE [20,21]. Additionally, it was also revealed that the molecular weight of lignin also influences its antioxidant activity [22].

Moreover, antiradical activity of lignin can be used in plastic films for packaging applications. For example, poly(lactic acid) (PLA) extruded films containing different amounts of lignin nanoparticles were investigated by determination of the radical scavenging activity of these composites [23]. Lignin nanoparticles proved to be highly efficient in terms of antioxidation activity, based on the disappearance of the absorption band at 517 nm of the free radical, 2,2-diphenyl-1-picrylhydrazyl (DPPH), upon reduction by an antiradical compound. Finally, migration results showed that PLA/lignin nanoparticle films can be considered suitable for application in the food packaging sector.

Lignin-based composites can be used as a UV barrier for food packaging films due to low permeation of UV light [24] and the antioxidant properties of lignin [25,26].

In the literature there are numerous information concerning the relation of lignin content vs. thermoplastics properties. On the other hand, some information regarding its technological properties is missing. From a practical point of view, this data can be crucial considering the real application of fillers based lignin for example in packaging, protection bags, containers, etc. Therefore, we are convinced that there is a necessity to investigate the technological properties of lignin/polyethylene bio composites.

In this research, materials including LDPE/MgO-lignin were manufactured by adding various composition of hybrid fillers into film grade low density polyethylene, in order to study the effects of lignin addition on the mechanical and technological properties of bio composites. Lignin used in this research was a pure alkali lignin (industrial kraft preparation process), mechanically coupled at different ratios with magnesium oxide. Maleic anhydride was introduced as a third additive to enhance the compatibility between components polyethylene-grafted. The effect of MgO-lignin content on the technological properties of the bio composites was studied by using weldability procedure and additionally the mechanical properties of the welds were measured. 

## 2. Materials and Methods

### 2.1. Materials

As part of this work, the following materials were used: magnesium oxide (Sigma-Aldrich, Steinheim am Albuch, Germany) and kraft lignin (Sigma-Aldrich, Germany). As a polymeric material low density polyethylene (LDPE) Malen E FGNX 23-D006, from BasellOrlen Polyolefins Sp. zo.o. (Płock, Poland), with its melt flow rate (MFR) of 0.8 g/10 min at 190 °C was used. As a compatibilizing agent polyethylene-graft-maleic anhydride (PE-g-MAH), provided by Sigma-Aldrich (Germany) with 0.5% by weight of maleic anhydride concentration, was used. The polar reagent was used in the amount of 2% by weight of total mass, and it’s based on our previous experiments with successful coupling of HDPE and zinc oxide-lignin dual filler with MAPE [16].

### 2.2. Preparation of MgO-Lignin Hybrid Systems

**Figure 1:**
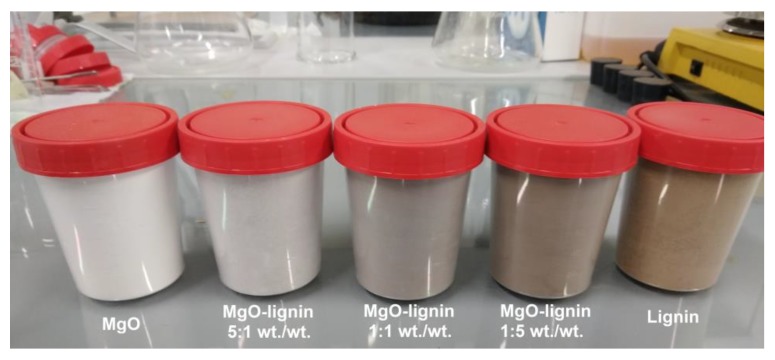
Digital photo of the obtained hybrid fillers.

MgO-lignin hybrids were obtained using magnesium oxide and kraft lignin. The final products in the form of MgO-lignin systems were prepared at three different ratos: (i) one part by weight of MgO for 5 parts by weight of lignin (1:5); (ii) one part by weight of MgO per one part by weight of lignin (1:1); (iii) five parts by weight MgO for one part by weight of lignin (5:1). Both components were mechanically connected by grinding and homogenizing the system in a planetary ball mill (Fritsch GmbH, Idar-Oberstein, Germany) for 2 h. A detailed description of the production methodology can be found in our previous publications [9,16,27,28]. Additionally, fillers in the form of pure magnesium oxide and pure lignin were prepared. After milling, the powders were sieved through a sieve with a diameter of 80 mm. Then, the samples were subjected to further tests. The received materials are presented on a digital photo (see Figure 1).

### 2.3. Physicochemical and Dispersive-Microstructural Characteristics of Hybrid Systems

Dispersive and microstructural properties were evaluated using scanning electron microscopy (SEM) and recorded using an EVO40 microscope (Zeiss, Jena, Germany). 

The particle size range was determined using a Mastersizer 2000 instrument (Malvern Instruments Ltd., Malvern, UK) by laser diffraction technique. Additionally, Zetasizer Nano ZS (Malvern Instruments Ltd.) was used to determine the particle size distributions, based on the noninvasive backscattering (NIBS) method.

The efficiency of obtaining hybrid materials was confirmed using Fourier transform infrared spectroscopy (FTIR). For this purpose, the Vertex 70 spectrometer (Bruker Optik GmbH, Ettlingen, Germany) was used. The designed hybrid materials and pure components were analyzed in the form of potassium bromide tablets. The FTIR analysis was performed at a wavenumber range of 4000–400 cm^−1^.

The thermogravimetric analysis (TGA) of the samples was determined using a Jupiter STA449F3 analyzer (Netzsch GmbH, Selb, Germany). Measurements were conducted at a heating rate of 10 °C/min over the temperature range of 25–600 °C under nitrogen flow (10 mL/min).

### 2.4. Preparation of Polyethylene/PE-g-MAH/MgO-Lignin Hybrid Composites

The processing of LDPE/PE-g-MAH/MgO-lignin hybrid composites was divided into two general steps. At first stage, all components were dehumidified with utilization of air-circulating oven (Memmert 500w type, Büchenbach, Germany) at 80 °C for 24 h. Dried polymers and fillers were subjected to compounding with twin screw extruder, Zamak 16/40 EHD (Skawina, Poland) under the processing parameters: barrel temperature of 160–195 °C and a screw rotation speed of 150 rpm. Applied twin screw extruder with the screws diameter of 16 mm and L/D ratio of 40, has a plasticizing unit with intensive shear zones, which were presented in detail in [9]. To prevent secondary moisturizing of hydrophilic filler, the extrudates were cooled down by using a cooling tunnel equipped with silicone belt and air fans, and finally pelletized.

At the second stage, the pristine LDPE and LDPE/PE-g-MAH/MgO-lignin composites were processed with a single screw extruder Metalchem 28/30 (Gliwice, Poland) where produced films were shaped by using a 1 mm thick and 250 mm long slit die, main cooled roller and two tracer rolls coated with rubber. The temperature of the extruder heating sections was set as: 160 °C, 175 °C, 180 °C, 185 °C, 190 °C, 200 °C, and screw rotation was set as 70 rpm. The temperature of main cooled roller was established as a 45 °C and maintained by the external thermostat, their speed was set as a 2 m/min. After manufacturing procedure, all of the produced films were conditioned at 23 °C, 50% RH for 48 h.

The prepared thin sheet films for all compositions were listed in Table 1.

### 2.5. Welding Conditions of Polyethylene/Hybrid Composites

An impulse, two heating bars welding machine was used for preparing of welded joints. The laboratory welding equipment has a two heating bars covered by release anti-adhesive fabric where the heating mode could be realised as a pulsed current flow. In order to adjust the heating time to make an uniform weld, films were firstly welded at different heating times, and then manually tested (the operator tried to break them). Welded joints were made at the lowest heating time ensuring a film break instead of a weld. Finally, the sealing parameters were selected: time of electric impulse: 2.0–3.0 s (depending on the compositions), cooling and compressing: 2.5 s.

The ultimate strength of the welds was estimated in accordance to ASTM F 88/F88M-09 (trouser test), lap shear test as well as tear test of welded joints. 

For mechanical tests, the joints were performed in stripes form with 15 mm width. For all tensile tests, the initial force of 2 N was applied. Mechanical tests were conducted using a ZwickRoel Z010 instrument (Kennesaw, GA, USA) with a 10 kN load cell at a crosshead speed of 200 mm/min. The grips were equipped with jaws inserts covered with soft PU to prevent strip damage in the mounting area. 

## 3. Results and Discussion

### 3.1. Dispersive-Morphological and Physicochemical Properties of MgO-Lignin Hybrid Materials

**Figure 2:**
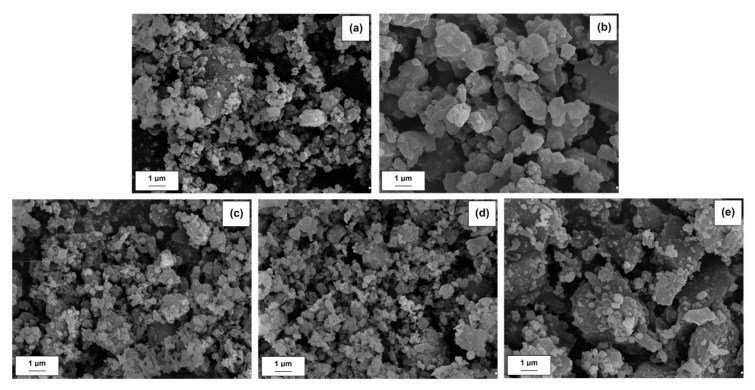
SEM images of MgO (**a**), lignin (**b**) and MgO-lignin hybrid materials with a weight ratio of components equal to: 5:1 *w*/*w* (**c**), 1:1 *w*/*w* (**d**), and 1:5 *w*/*w* (**e**).

SEM images for pure components (MgO and lignin) as well as hybrid materials obtained by mechanical means are presented in Figure 2. A SEM photo of pure MgO (see Figure 2a) indicates the presence of single primary particles which tend to form aggregate and agglomerate forms. In turn, lignin is characterized by the presence of larger particles with irregular shape (see Figure 2b). The designed hybrid materials are characterized by a different structure, which depends on the composition of individual components. It can be seen that as the biopolymer content increases, the particle size increases and the systems become less homogeneous.

The particle size results measured using Mastersizer 2000 (see Table 2) confirm the conclusions obtained from the analysis of SEM images. 

The average particle size of MgO, determined using the Mastersizer 2000, is 1.5 µm. Lignin is characterized by much lower homogeneity. In addition, the average particle size is definitely higher than the inorganic component and amounts to 6.4 µm. Analysis of the obtained results for hybrid materials clearly indicates that as the lignin content increases, the systems are less homogeneous and show the presence of larger particles. The average particle size for MgO-lignin hybrid systems, depending on the ratio of the components used (5:1 *w*/*w*, 1:1 *w*/*w*, and 1:5 *w*/*w*) is equal to 2.9 µm, 3.3 µm, and 4.2 µm, respectively. However, these results do not exclude the possibility to further enhance the properties of the composites.

**Figure 3:**
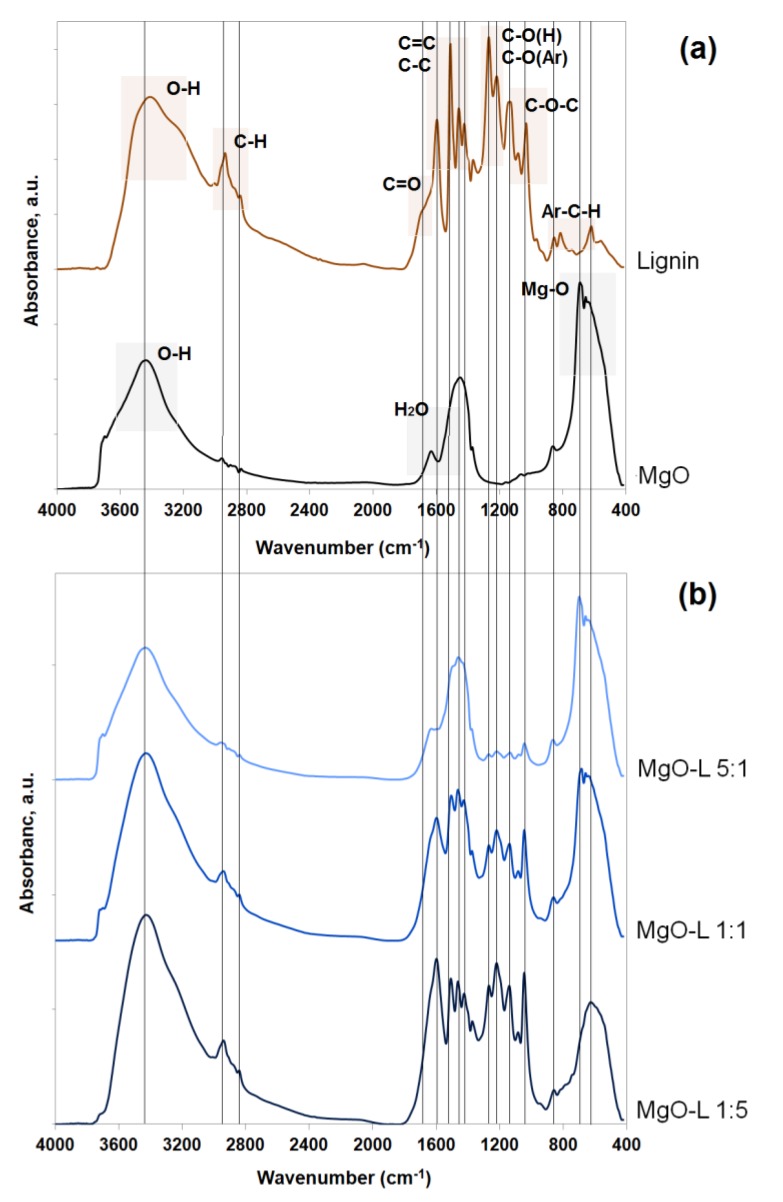
FTIR spectra of MgO and lignin (**a**) and MgO-lignin hybrid materials (**b**).

The efficiency of obtaining MgO-lignin hybrid materials was confirmed using Fourier transform infrared spectroscopy (see Figure 3).

Magnesium oxide is characterized by the presence of stretching vibrations of hydroxyl group bonds, with a maximum absorption recorded at a wavenumber of 3430 cm^−1^. In addition, the ~1610 cm^−1^ band is associated with the presence of water physically bound to the oxide surface. The most important band which confirms the presence of Mg-O bonds is the band with the maximum occurring at wavenumber of 673 cm^−1^.

The second of the components of the hybrid system, lignin, is characterized by the presence of stretching vibrations of O-H bonds at the maximum wavenumber of 3405 cm^−1^ and stretching vibrations of C-H bonds (CH3 and CH2—maximum at the wavenumber of 2890 cm^−1^ and O-CH3—2815 cm^−1^). Important groups of bonds present in the lignin structure are: C=O (1670 cm^−1^), C-C and C=C (associated with the aromatic skeleton of the biopolymer) with a maximum of wavenumbers respectively equal to 1598 cm^−1^, 1552 cm^−1^, 1518 cm^−1^, 1482 cm^−1^, and 1443 cm^−1^, followed by C-O(H) and C-O(Ar) at a maximum of 1251 cm^−1^ and 1208 cm^−1^ as well as C-O-C associated with the bands present at a maximum of wavenumbers of 1094 cm^−1^ and 1015 cm^−1^. These data are consistent with those published previously [9,16,27,29].

The efficiency of obtaining hybrid materials is primarily indicated by the presence of bands associated with individual components in final systems. Unstable hydrogen bonds have formed between the components, which is confirmed by small shifts of individual absorption bands of functional groups. These bonds are sufficient for the use of such systems as polymer fillers. Thus, in this case, class I hybrid material is obtained with physical interactions between the components. A similar situation was observed in our earlier publications, in which SiO_2_-lignin [9,17,27], Al_2_O_3_-lignin [30], or ZnO-lignin [16,28] hybrid materials were produced.

**Figure 4:**
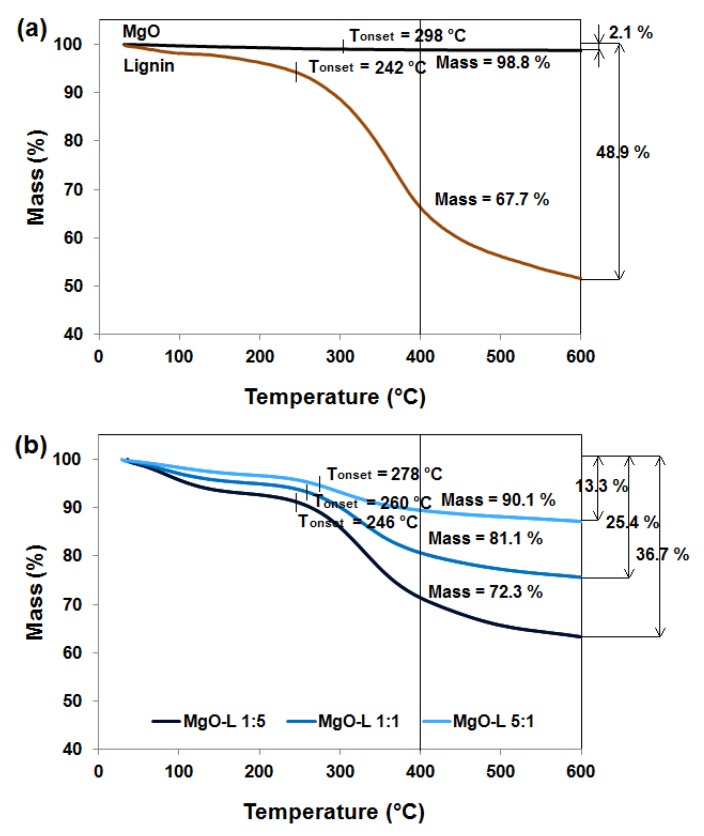
TGA curves of MgO and lignin (**a**) and MgO-lignin hybrid materials (**b**).

Assessment of thermal stability is an important analysis carried out in order to determine the effect of the filler on the properties of polymer composites. Therefore, thermogravimetric curves for MgO, lignin, and obtained MgO-lignin hybrid materials is presented in Figure 4.

Lignin has very limited thermal stability and loses almost 50% of its initial mass at temperature up to 600 °C. The biopolymer is also characterized by three main stages of mass loss and we have described it extensively in earlier publications [9,27,28,31,32]. Therefore, in order to improve the thermal stability of the biopolymer, in this study was used magnesium oxide which is very resistant to high temperatures and loses 2.1% of its mass at a given temperature.

Hence, the obtained hybrid materials exhibit fairly good thermal stability, which improves as the content of the inorganic component increases. The total weight loss at the analyzed temperature for MgO-lignin hybrid materials with a component ratio of 5:1 *w*/*w*, 1:1 *w*/*w*, and 1:5 *w*/*w* is equal to 13.3%, 25.4%, and 36.7%, respectively. Additionally, the corresponding sample mass at 400 °C and the initial degradation temperature (Tonset) for each are marked in Figure 4. Similar relationships regarding the thermal stability of hybrid materials based on lignin were obtained for SiO_2_-lignin [9,17,27] or ZnO-lignin [16,28] hybrid systems.

### 3.2. Technological Properties of Welded Polyethylene/Hybrid Composites

#### 3.2.1. Weldability of Polyethylene/Hybrid Composites

As presented in Table 3, the weldability experiments showed that initial heating time set as 2 s during preliminary attempts was insufficient to obtain correctly welded joints for the LDPE/MgO and LDPE/MgO-L (5:1 *w*/*w*) composites. Therefore, the heating time for these composites was adjusted to 3.0 s and 2.5 s, respectively. The heating time correction could be linked with a shift of the softening point for LDPE/MgO composition (possible explanation) or with the increase of thermal conductivity of the composites, which contained a high amount of magnesium oxide. 

**Figure 5:**
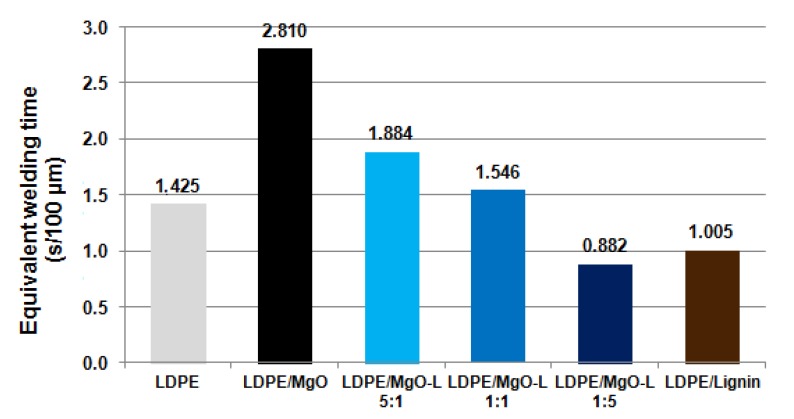
Required heating time of the tested films with relation to its thickness.

Data presented in Table 3 are not adequate to assess the weldability of studied materials, because of the differences in film thickness that was used for estimation of total heating time. Therefore, an equivalent welding time is a more realistic parameter, which takes into account the time consumed for heating the film with normalized thickness, which was defined as 100 µm. Final results regarding the recalculated welding time are presented in Figure 5. 

The results showed that equivalent time needed to produce correct welds has lower value for composites in which the highest amount of lignin was used in hybrid filler. In other words, in order to seal the package, the hybrid filler MgO-L (1:5 *w*/*w*), will be the most appropriate additive into LDPE films. The results can be connected with dispersibility of fillers in polyethylene matrix [17]. 

#### 3.2.2. Seal Force Measurement of Polyethylene/Hybrid Composites

**Figure 6:**
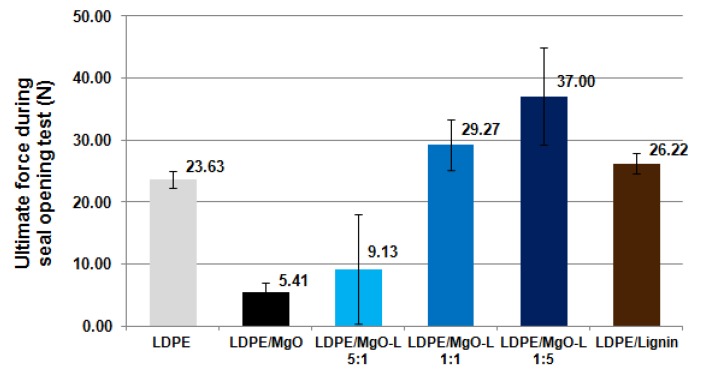
Comparison of the seal open force for tested materials.

For films packaging application, seal force/strength has to be measured in order to estimate the performance of the seal joint. Seal force testing, also known as peel testing, deals with the determination of the strength of non-rigid film seals. Seal strength is a quantitative measure for use in process validation, process control and capability. Seal strength procedure could be also applied to measure the ability of the packaging processes to produce consistent seals. The results presented in Figure 6, clearly indicate that MgO coupled with a high lignin content may promote seal open force markedly higher than in the case of the original, non-filled LDPE. On the other hand, for composites with MgO used as a filler, most of the specimens possessed a broken inside welding seam, which is an incorrect behaviour for a sealed package.

#### 3.2.3. Shear Test of Welded Joint of Polyethylene/Hybrid Composites

**Figure 7:**
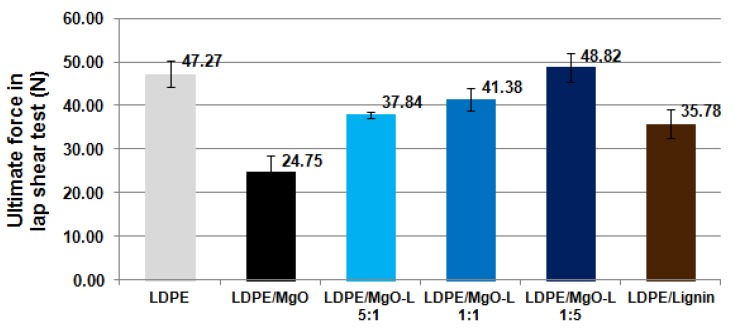
Comparison of the ultimate shear force applied in lap shear test.

Lap shear test describes the situation in which the weld is loaded by the force applied transverse to the weld plane. The way of specimen mounting is viewed in Figure 9b. In such case, tested composites exhibited a decrease of the ultimate force compared to neat LDPE, except the composition with MgO-L (1:5 *w*/*w*), as can be seen in Figure 7. 

For this material, the mean value of the recorded breaking force which was noted as 48.8 N is only slightly higher than the adequate force for LDPE, which was recorded as 47.3 N. These results may indicate that during the lap shear test, the uniform structure of weld (less interphases in material structure) allows to obtain a stronger weld, in case of neat LDPE. On the other hand, the best weldability checked previously for the composite with the highest biopolymer content in hybrid filler may cause some positive effects in the distribution of shear stresses during weld shearing.

#### 3.2.4. Tear Test of Welded Films of Polyethylene/Hybrid Composites

**Figure 8:**
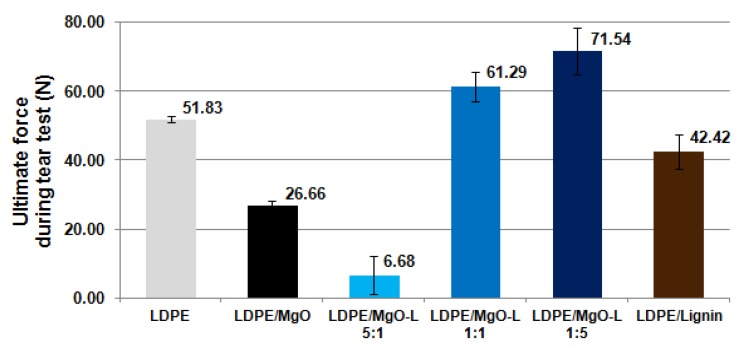
Comparison of the ultimate shear force applied in lap shear test.

**Figure 9:**
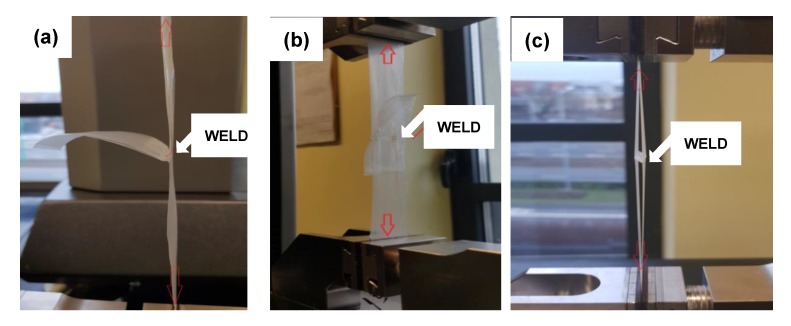
View of the welded specimens mounted in tensile machine grips, in tested mode: (**a**) seal strength, (**b**) lap shear test, and (**c**) tear the weld test.

The results of tear test of welded films are presented in Figure 8, and the way of mounting the specimen with the grips is presented in Figure 9c. Once again, the use of dual fillers has significantly increased tear resistance of prepared weld, except for MgO-L (5:1 *w*/*w*). The use of lignin as a filler resulted in a decrease of force at break by approx. 10 N compared to neat LDPE. The composites with MgO and hybrid MgO-L (5:1 *w*/*w*) fillers completely failed the tear test, as the mean values of tear resistance decreased by about 25 N and 45 N, respectively, compared to the base material. Those results are in good relation with the lowest efficiency of weldability, which corresponds to the highest equivalent welding time.

Figure 9 present the pictures of selected specimens during mechnical tests with different methods. It should be noted, that the testing procedure should be carried out with an appropriate mount the specimens in one axe, on the other hand, because of the samples are not rigid, the initial force should be applied before starting the main load.

## 4. Conclusions

In the framework of this work, we tested a new type of partially bio-based fillers, hybridized by mechanically coupled magnesium oxide with kraft lignin particles. As a result, we obtained class 1 hybrid fillers in which physical interactions occur in the form of hydrogen bonds. The inorganic component, in the form of magnesium oxide, has significantly improved the thermal stability of the final hybrid systems. In addition, it should be noted that the newly designed materials are characterized by fairly good homogeneity, which was confirmed by the obtained dispersion parameters data and SEM images.

Experiments showed that the application of novel, partially bio-based fillers into LDPE may significantly change the technological parameters of prepared films by means of welding conditions and also improve some mechanical parameters of the welds.

The applied welding procedure related to determination of the minimum heating time for the weld confirmed that partially bio-based magnesium oxide-lignin dual phase fillers reduce the time and, in fact, energy consumption needed to seal the film (packaging). Equivalent heating time of composites with a hybrid filler which contained 80% amount of lignin in total weight of filler was about 40% shorter than in the case of films extruded from neat LDPE.

The mechanical test showed that the addition of dual fillers based on magnesium oxide and lignin increases the weld strength of composites, shear strength, and the force needed for tear the weld. Moreover, the effect of increasing strength occurs only with the increase of the lignin concentration in the dual phase filler. This observation clearly confirms the positive role of lignin and its coupling with MgO in case of LDPE modification. The practical use of such systems may include packaging materials or films for agricultural application. 

## Figures and Tables

**Table 1:** The list of prepared compositions with content of MgO, lignin and MgO-lignin fillers in the LDPE matrix in extruded films.

Film Composition	Composition			
	Polymer Content (%) of Weight	Filler Content (%) of Weight	PE-g-MAH (%) of Weight	Chill Roll Speed (m/min)
LDPE	100	–	–	2.0
LDPE/MgO	93	5.0	2.0	2.0
LDPE/MgO-L (5:1 *w*/*w*)				
LDPE/MgO-L (1:1 *w*/*w*)				
LDPE/MgO-L (1:5 *w*/*w*)				
LDPE/Lignin				

**Table 2:** Particle diameter of MgO, biopolymer and MgO-lignin hybrids from Mastersizer 2000.

Sample Name	Particle Diameter from Mastersizer 2000 (μm)			
	d(0.1) ^1^	d(0.5) ^2^	d(0.9) ^3^	D[4.3] ^4^
MgO	0.6	1.2	2.2	1.5
Lignin	2.0	5.1	8.3	6.4
MgO-L (1:5 *w*/*w*)	1.5	4.0	4.6	4.2
MgO-L (1:1 *w*/*w*)	1.4	3.1	4.3	3.3
MgO-L (5:1 *w*/*w*)	1.3	2.8	4.2	2.9

^1^ d(0.1)—10% of the volume distribution is below this value diameter; ^2^ d(0.5)—50% of the volume distribution is below this value diameter; ^3^ d(0.9)—90% of the volume distribution is below this value diameter; ^4^ D[4.3]—average particle size in examined system.

**Table 3:** Welding conditions applied to achieve welded joint of extruded films.

Film Composition	Mean Film Thickness (mm)	Adjusted Heating Time during Welding (s)
LDPE	0.140	2.0
LDPE/MgO	0.107	3.0
LDPE/MgO-L (5:1 *w*/*w*)	0.133	2.5
LDPE/MgO-L (1:1 *w*/*w*)	0.129	2.0
LDPE/MgO-L (1:5 *w*/*w*)	0.227	2.0
LDPE/Lignin	0.199	2.0
